# Investigating Biological Control Agents for Controlling Invasive Populations of the Mealybug *Pseudococcus comstocki* in France

**DOI:** 10.1371/journal.pone.0157965

**Published:** 2016-06-30

**Authors:** Thibaut Malausa, Mathilde Delaunay, Alexandre Fleisch, Géraldine Groussier-Bout, Sylvie Warot, Didier Crochard, Emilio Guerrieri, Gérard Delvare, Giuseppina Pellizzari, M. Bora Kaydan, Nadia Al-Khateeb, Jean-François Germain, Lisa Brancaccio, Isabelle Le Goff, Melissa Bessac, Nicolas Ris, Philippe Kreiter

**Affiliations:** 1 INRA, Univ. Nice Sophia Antipolis, CNRS, UMR 1355–7254 Institut Sophia Agrobiotech, 06900, Sophia Antipolis, France; 2 Istituto per la Protezione Sostenibile delle Piante, Consiglio Nazionale delle Ricerche, Via Università 133, 80055 Portici, Napoli, Italy; 3 CIRAD, UMR CBGP INRA CIRAD Montpellier Supagro, 755 avenue du Campus Agropolis, CS 30016, 34988, Montferrier-sur-Lez Cedex, France; 4 Università di Padova, Dipartimento Agronomia, Animali, Alimenti, Risorse naturali e Ambiente, Viale dell’Università 16, 35020, Legnaro, Italy; 5 Imamoglu Vocational School, Çukurova University, Adana, 01330, Turkey; 6 Lattakia Center for Rearing Natural Enemies, Lattakia, Syria; 7 ANSES, Laboratoire de la Santé des Végétaux, Unité d’entomologie et Plantes Invasives 755 avenue du Campus Agropolis, CS 30016, Montferrier-sur-Lez, France; University of Pretoria, SOUTH AFRICA

## Abstract

*Pseudococcus comstocki* (Hemiptera: Pseudococcidae) is a mealybug species native to Eastern Asia and present as an invasive pest in northern Italy and southern France since the start of the century. It infests apple and pear trees, grapevines and some ornamental trees. Biocontrol programmes against this pest proved successful in central Asia and North America in the second half of the 20^th^ century. In this study, we investigated possible biocontrol agents against *P*. *comstocki*, with the aim of developing a biocontrol programme in France. We carried out systematic DNA-barcoding at each step in the search for a specialist parasitoid. First we characterised the French target populations of *P*. *comstocki*. We then identified the parasitoids attacking *P*. *comstocki* in France. Finally, we searched for foreign mealybug populations identified *a priori* as *P*. *comstocki* and surveyed their hymenopteran parasitoids. Three mealybug species (*P*. *comstocki*, *P*. *viburni* and *P*. *cryptus*) were identified during the survey, together with at least 16 different parasitoid taxa. We selected candidate biological control agent populations for use against *P*. *comstocki* in France, from the species *Allotropa burrelli* (Hymenoptera: Platygastridae) and *Acerophagus malinus* (Hymenoptera: Encyrtidae). The coupling of molecular and morphological characterisation for both pests and natural enemies facilitated the programme development and the rejection of unsuitable or generalist parasitoids.

## Introduction

*Pseudococcus comstocki* (Hemiptera: Pseudococcidae) is a mealybug species native to Eastern Asia that has been present as an invasive pest in northern Italy and southern France since the turn of the century [1, 2], infesting apple, pear and ornamental trees of the genera *Morus* and *Catalpa* in particular. *P*. *comstocki* causes economic losses, principally due to a decrease in fruit marketability as a consequence of the massive development of sooty mould on the honeydew excreted by the mealybug which accumulates on the leaves and fruits. For example, in several infested orchards in southern France up to 80% of the harvested fruits are discarded when infestation rates are high and accumulation of sooty mould is particularly severe on the surface or the carpel of the fruits (Philippe Kreiter, 2009, pers. com.). The presence of *P*. *comstocki* populations also weakens the plants in other ways, through sap-feeding and virus transmission [3].

Biological control is often used as an alternative to pesticides for the control of mealybugs [4]. This approach has repeatedly proven to be successful and safe for non-target organisms [5, 6]. Specific hymenopteran parasitoids have been used to control *P*. *comstocki*, including *Acerophagus* (= *Pseudaphycus*) *malinus* (Hymenoptera: Encyrtidae) in the former USSR [7–12] and in the USA [13, 14], and *Allotropa burrelli* (Hymenoptera: Platygastridae) in the USA [14, 15].

In 2008, the French National Institute of Agronomy (INRA) set up a classical biological control programme for *P*. *comstocki* in France. This programme began with a study of the French populations of *P*. *comstocki* and a survey of the natural enemies associated with *P*. *comstocki* in France. From 2009 to 2011 we looked for *P*. *comstocki* populations abroad (Italy, Syria, China, Japan, Turkey) and surveyed their natural enemies. The first samples of exotic material were imported in 2010 from Japan and the first releases of natural enemies took place in 2012.

It has been repeatedly argued and observed that classical biological control benefits from the use of molecular tools [16–19]. DNA-based methods help to identify the target pest and its possible natural enemies with precision in the native area and in areas in which biocontrol is considered. This makes it possible to avoid (i) a mismatch between the target pest and the biological control agent released, and (ii) the choice of a natural enemy with highly generalist behaviour in the native area that might have unintended effects on local biodiversity in the region in which it is released. The use of DNA techniques to characterise the biological material used throughout the biocontrol programme also makes it possible to implement quality and traceability procedures, which may be required to obtain governmental authorisation for the importation of biological material.

At each step in the *P*. *comstocki* biological control programme, we made use of molecular characterisation methods, the results of which helped to guide key decisions. We first used DNA sequencing to characterise French *P*. *comstocki* populations, by methods recently used for the DNA barcoding of mealybugs [20–23]. We then used molecular and morphological methods to characterise the parasitoids present in these mealybugs in France. The next step was the search for exotic candidate biocontrol agents. We used DNA sequencing to check that the foreign mealybugs identified as *P*. *comstocki* did indeed have DNA sequences similar to those of the French *P*. *comstocki* populations. We then used DNA sequencing and morphological examination to characterise and identify as accurately as possible all of the parasitoids collected from the foreign *P*. *comstocki* populations.

We present here (i) an overview of the classical biocontrol programme, which resulted in the field release of two natural enemies: augmentative biological control using French populations of *A*. *malinus*, and the introduction of exotic *A*. *burrelli* populations, (ii) the methods and results of the molecular and morphological characterisations carried out at each step in the classical biocontrol programme, and (iii) a discussion of the added value provided by the molecular identification methods.

## Materials and Methods

### Description of the biocontrol programme and the sampling strategy

The biological control programme was initiated in 2008, following the detection of *P*. *comstocki* in apple orchards in southern France in 2005 [1]. The programme was coordinated by INRA (France) and focused on orchards located along the French Mediterranean coast and in the southern Rhone valley.

From 2008 to 2010, populations of *P*. *comstocki* and its natural enemies were sampled in the field in France. The material collected was used for morphological and DNA-based identification [24]. In total, 16 mealybug population samples were collected (Table 1), together with the parasitoids emerging from them. The collected mealybugs and parasitoids were conserved in 70% to 96% ethanol and stored at -20°C for further characterisation.

**Table 1:** Sampling sites: code, host plant, collection date, country, region and site of sampling, number of individuals with DNA data: mealybugs with sequence data, mealybugs with rapid PCR identification data, most likely mealybug species present based on molecular data (between *P*. *comstocki*–PC—and *P*. *viburni–*PV which were often encountered in sympatry), parasitoids with DNA sequence data.

Site code	Host plant	Date	Country	County	Site / City	N sequenced mealybugs	N PCR identified mealybugs	Most likely host (PC, PV)	N sequenced parasitoids
C01	*Vitis vinifera*	07/2010	China	Shangdong	Shangdong	6	-	PC?	-
F01	*Morus kagayamae*	2009–2010	France	Var	Roquebrune-sur-Argens	7	-	PC	71
F02	*Malus pumila*	08/2008	France	Gard	Beaucaire	-	5	PV	3
F03	*Malus pumila*	09/2008	France	Gard	Manduel	-	7	PC(1),PV(6)	1
F04	*Malus pumila*	09/2008	France	Hérault	Marsillargues	-	13	PC(12),PV(1)	26
F05	*Malus pumila*	07-10/2008	France	Hérault	Mauguio	-	-	PC/PV	4
F06	*Malus pumila*	08/2007	France	Hérault	Montpellier	-	5	PC	4
F07	*Malus pumila*	07-09/2008	France	Gard	Rodilhan	-	-	PC/PV	2
F08	*Morus kagayamae*	07-09/2008	France	Bouches du Rhône	Saintes-Maries-de-la-Mer	-	20	PC	26
F09	*Malus pumila*	07/2008	France	Gard	Saint-Geniès-des-Mourgues	-	-	PC/PV	1
F10	*Malus pumila*	09/2008	France	Gard	Saint-Gilles	-	7	PC	42
F11	*Malus pumila*	07-09/2008	France	Bouches du Rhône	Sambuc	-	-	PC/PV	5
F12	*Malus pumila*	08/2011	France	Bouches du Rhône	Mallemort	-	5	PC	4
F13	*Malus pumila*	07-09/2008	France	Gard	Jonquières-Saint-Vincent	-	4	PC(3),PV(1)	-
F14	*Malus pumila*	09/2008	France	Gard	Fourques	-	9	PV	-
F15	*Malus pumila*	08/2008	France	Gard	Caissargues		7	PC(1),PV(6)	-
F16	*Solanum tuberosum* (lab)	12/2010	France	Alpes-Maritimes	Sophia Antipolis	5	-	PC	-
F17	*Malus pumila*	08/2010	France	Gard	Bellegarde	3	-	-	-
I01	*Viburnum tinus*	09/2009	Italy	Veneto	Treviso	6	-	PV	-
I02	*Morus nigra*	09/2009	Italy	Veneto	Dossobuono	4	-	PC	10
I03	*Prunus persica*	09/2009	Italy	Veneto	Alpo di Villafranca	4	-	PC	-
I04	*Catalpa sp*.	09/2009	Italy	Veneto	Castelnuovo del Garda	5	-	PC	1
J01	*Pyrus sp*.	08/2010	Japan	Tottori	Tottori	5	-	PC	12
J02	*Vitis sp*.	08/2010	Japan	Izumo	Izumo	15	-	PC	20
J03	*Malus sp*.	08/2010	Japan	Utsunomiya	Utsunomiya	6	-	PC	3
J04	*Solanum tuberosum* (lab)	08/2010	Japan	Utsunomiya	Utsunomiya	2	-	PC	-
S01	*Citrus sp*.	09/2009	Syria	Latakia	Latakia	60	-	-	38
T01	*Morus sp*.	2010	Turkey	Igdir	Igdir	4	-	PC	10
T02	*Punica granatum*	2010	Turkey	Artvin	Artvin	16	-	PC	32

From 2009 to 2011, we sampled and characterised exotic populations of *P*. *comstocki* and their associated natural enemies (Table 1) in regions where the occurrence of *P*. *comstocki* had been recorded recently. The first samples were obtained from Syria and Italy in 2009. In 2010, samples were obtained from China and Japan. In 2011, other populations were sampled in eastern Turkey. In total, 14 populations of mealybugs were collected outside France (Table 1). Parasitoids were collected from infested mealybugs in Syria, Italy, Japan and Turkey (Table 1). The samples of mealybugs and parasitoids were conserved in 70% to 96% ethanol and stored at -20°C for further characterisation (“Characterisation of mealybug and parasitoid material” section).

### Characterisation of mealybug and parasitoid material

#### Mealybug DNA extraction, amplification and sequencing

For each individual (whatever its development stage), genomic DNA was extracted with the DNeasy Tissue Kit (QIAGEN, Hilden, Germany). Animals were not crushed before extraction. Instead, we extended the cell lysis time beyond that recommended by the manufacturer (6–8 h, rather than the recommended 4–6 h). Two elution steps were performed with AE buffer to increase the amount of DNA extracted: 2 x 20 μL for the smallest mealybugs (L1), 2 x 30 μL for L2 and L3, 2 x 50 μL for adults.

Three DNA regions were studied for the mealybug samples: 28S, ITS2 and the LCO region of the cytochrome oxidase subunit I (COI) gene (hereafter referred to as M-28S, M-ITS2 and M-LCO, respectively). PCRs for the M-28S and M-ITS2 markers was performed with the Phusion High-Fidelity DNA polymerase 530L (FINNZYMES, Espoo, Finland). PCR for the M-LCO marker was performed with the Qiagen Multiplex PCR kit (QIAGEN, Hilden, Germany). All PCRs were performed in a total volume of 25 μL: 23 μL of mix + 2 μL of diluted DNA (between 1 and 20 ng). For M-28S and M-ITS2, the reagent concentrations were: 1x Phusion HF buffer, 0.01 U/μL Phusion enzyme, 200 μM dNTPs and 0.5 μM of each primer. For M-ITS2, we added 1.5 mM MgCl_2_ and 6% DMSO. For M-LCO, the reagent concentrations were: 12.5 μL Qiagen buffer and 0.2 μL of each primer in the total volume. All the primers used are listed in Table 2.

**Table 2:** List of the markers used in the PCR protocols: organism (mealybug or parasitoid), name of the target region, name of primers, 5’–3’ primer sequence, reference from which the sequences were obtained.

Organism type	Locus name	Primer name	F/R	Primer sequence	References
Mealybugs	M-28S	M28S-F	F	AGAGAGAGTTCAAGAGTACGTG	Belshaw and Quicke [36]
		M28S-R	R	TTGGTCCGTGTTTCAAGACGGG	
	M-LCO	MLCOn-F	F	AYAATATAATRATTACWWTWCATGC	Abd-Rabou *et al*. [22]
		MLCOn-R	R	TTTWCCATTTAAWGTTATTATTC	
	M-ITS2	MITS2-F	F	CTCGTGACCAAAGAGTCCTG	Malausa *et al*. [20]
		MITS2-R	R	TGCTTAAGTTCAGCGGGTAG	
Parasitoids	P-28S	P28S-F	F	CGTGTTGCTTGATAGTGCAGC	Heraty *et al*. [37]
		P28S-R	R	TCAAGACGGGTCCTGAAAGT	
	P-C1	PC1-F	F	CAACATTTATTTTGATTTTTTGG	Simon *et al*. [38]
		PC1-R	R	TCCAATGCACTAATCTGCCATA	
	P-LCO	LCO	F	GGTCAACAAATCATAAAGATATTGG	Folmer *et al*. [39]
		HCO	R	TAAACTTCAGGGTGACCAAAAAATCA	
	P-ITS2	PITS2-F	F	GGGTCGATGAAGAACGCAGC	Navajas *et al*. [40]
		PITS2-R	R	ATATGCTTAAATTCAGCGGG	

For M-28S and M-ITS2, PCR conditions were: initial denaturation at 98°C for 30 s, followed by 35 cycles of (i) denaturation at 98°C for 10 s, (ii) annealing at 58°C for 15 s, (iii) elongation at 72°C for 15 s and a final extension period at 72°C for 5 minutes. For M-LCO, the PCR conditions were: initial denaturation at 95°C for 15 min, followed by 35 cycles of (i) denaturation at 95°C for 30 s, (ii) annealing at 48°C for 90 s, (iii) elongation at 72°C for 90 s and a final extension period at 72°C for 10 minutes.

PCR products were screened with the Qiaxcel Advanced system (QIAGEN, Hilden, Germany), with Fast Analysis cartridges (DM80 protocol). PCR products were then sent to Genoscreen (Lille, France) or Beckman Genomics (Takeley, United Kingdom) for bidirectional Sanger sequencing. Consensus sequences were generated and checked with Seqscape v2.7 (ABI). Alignments were edited manually with Bioedit 7.01 [25]. Sequences were deposited in Genbank.

#### Morphological examination of mealybugs

When possible, a few adult mealybug females per multi-locus haplotype determined by DNA sequencing were prepared for morphological examination, following the procedure described in Malausa et al. [20]. Briefly, mealybugs were prepared as follows: (i) the specimen was heated (≤ 40°C) in 10% KOH for 20 minutes; (ii) it was then rinsed in distilled water for 20 minutes; (iii) it was stained by incubation for one hour in a saturated solution of fuchsin in a 1:1:1 mixture of distilled water, lactic acid and glycerol; (iv) the specimen was washed in glacial acetic acid for one hour to stabilise the staining; (v) the specimen was transferred to lavender oil for at least one hour, placed in a drop of Canada balsam on a slide and covered with a coverslip. The slide was then labelled and observed under a microscope. In most cases, identification was based on the keys of Beardsley [26], Cox [27], Williams & Watson [28], Williams & Granara de Willink [29] and Williams [30].

The reference slide-mounted specimens used were obtained from the ANSES collection stored at the *Laboratoire de la Santé des végétaux*, *Unité d’entomologie et plantes invasives* (Montferrier-sur-Lez Cedex, France).

#### Parasitoid DNA extraction, amplification and sequencing

For each adult parasitoid, genomic DNA was extracted with the Prepgem Insect kit (Zygem, Hamilton, New Zealand). Parasitoids were not crushed before extraction and the time period over which the Prepgem enzyme was allowed to act was extended beyond the manufacturer’s recommendations (2 h rather than 30 minutes). The total volume of 1X Prepgem Buffer and enzyme used was 30 μL per individual.

Four DNA regions were studied: 28S, ITS2 and two regions of the cytochrome oxidase subunit I gene (hereafter referred to as P-28S, P-ITS2, P-LCO and P-C1, respectively). The primers used for each region are listed in Table 2. PCR was performed with the Qiagen Multiplex PCR Kit (QIAGEN, Hilden, Germany), with a reaction mixture of the same composition as for the mealybugs. PCR conditions were as follows: initial denaturation at 95°C for 15 minutes, followed by 35 cycles of (i) denaturation at 95°C for 30 s, (ii) annealing for 90 s at 54°C, 56°C, 48°C and 48°C for P-28S, P-ITS2, P-C1 and P-LCO, respectively, (iii) elongation at 72°C for 90 s, followed by a final extension period at 72°C for 10 minutes.

PCR products were screened with the Qiaxcel Advanced system (QIAGEN, Hilden, Germany), sequenced and analysed using the same methods as those used for mealybugs.

A neighbour-joining (NJ) tree was generated based on the number of nucleotide differences between 28S sequences, with Mega4 [31], in order to provide a visual representation of the data obtained for Encyrtidae parasitoids (this tree is not to provide phylogenetic information).

#### Morphological examination of parasitoids

Each of the parasitoids preserved in 70% to 96% ethanol was initially assigned to a morphospecies.

Card mounting: when available, at least five adult females and five adult males of each morphospecies were dried by placing them for 24 h in a 1:1 mixture of absolute ethanol-xylene. Specimens were then transferred to amyl acetate for 24 h and were rinsed in amyl acetate until the solvent completely evaporated. The dry specimens were mounted on card with water-soluble glue.

Slide mounting: when available, one male and one female from each morphospecies were selected from card-mounted material and processed as described by Noyes [32]. In brief, wings were dissected and mounted in a drop of Canada balsam, and the rest of the insect was detached from the card by applying a drop of distilled water, incubated with 10% KOH 100°C for five minutes and then with acetic acid at room temperature for 5 minutes. It was then dehydrated in a progressive series of ethanol solutions (concentrations from 70 to 100%). A drop of clove oil was added to the specimen in absolute ethanol, and the ethanol was allowed to evaporate off completely. Head, mouthparts, antennae, thorax, hypopygium (for females only) and genitalia were dissected and mounted in Canada Balm. Slide-mounted voucher specimens were deposited in the collection of the laboratory of Entomology “E. Tremblay”, Department of Agriculture University of Naples “Federico II”, Italy.

Identification of species was performed by comparing the material with type specimens and authoritatively identified material preserved at the Natural History Museum of London, UK, which houses the largest and best preserved collection of Hymenoptera parasitoids in the world.

### Assignment of the parasitoid host species

At least one of the mealybugs collected from each site outside France was systematically subjected to DNA sequencing. In France, during the parasitoid survey, only a small proportion of the mealybugs were identified by DNA sequencing, to decrease costs. Indeed, a rapid identification method based on species-specific PCR (Correa *et al*. in preparation) was used for mealybugs collected at sites located in regions for which DNA sequence data were already available (Table 1). The assignment of each parasitoid to a host was not 100% reliable, as identification was performed at population rather than individual level. We therefore did not necessarily identify the mealybug from which each parasitoid emerged. Outside France, only one mealybug species was found to be present at each sampling site, suggesting that host assignment was probably reliable. In France, *P*. *viburni* and *P*. *comstocki* often occurred together. Hence, in France, the reliability of host assignment was proportional to the number of mealybugs identified.

## Results

### Comparisons between invasive French *P*. *comstocki* populations and other populations worldwide

In total, we obtained 152 DNA sequences for M-28S, 144 for M-ITS2, 83 for M-LCO. The *P*. *comstocki* individuals collected in France had the M-28S-1, M-ITS2-1/2 and M-LCO-1 haplotypes (Table 3, including Genbank accession numbers).

**Table 3:** Haplotypes identified for the mealybugs collected in this study: most probable identification based on morphology and molecular data, haplotypes at 28S, ITS2, COI-LCO (and the corresponding NCBI genbank accession numbers), specimen codes of the individuals identified and codes of the sites at which they were collected.

Identification	Haplotypes			Individual sample codes	Sites
	28S	ITS2	LCO		
*Pseudococcus comstocki*	M28S-01 (KU499441)	MITS2-01 (KU499508), MITS-02 (KU499509)	MLCO-01 (KU499444), MLCO-02 (KU499445)	1383–1386, 1632, 1634, 1635, 1639, 1640, 1645, 5723, 5724, 5726, 5727, 5729, 5730, 5732, 5735–5738, 5741–5744, 5746, 5748–5752, 5754, 5960–5963, 6012–6015, 6019, 6022–6024, 6032, 6033, 8386, 8388–8390, 8392, 8397–8400, 8403, 8405–8408, 8410, 8411, 8413–8415, 9100–9103, 9110, 9111, 9113	C01, F01, F16, F17, T01-T02,
*Pseudococcus cryptus*	M28S-02 (KU499442)	MITS2-03 (KU499510)	MLCOn-03 (KU499446)	1022–1044, 1054–1061, 1305–1310, 1313, 1494–1508, 1513–1516, 1602, 1603, 1605, 8417–8420, 8422–8424	S01
*Pseudococcus viburni*	M28S-03 (KU499443)	MITS2-04 (KU499511)	MLCOn-04 (KU499447)	1623, 1625–1627, 9109, 9114–9116	F02-F04

Most of the samples collected in Italy were identified morphologically as *P*. *comstocki* (Table 3) and were found to display the same haplotypes as the French *P*. *comstocki* populations. At one site, mealybugs considered *a priori* to belong to the species *P*. *comstocki* were identified as *P*. *viburni*, with haplotypes M-28S-3, M-ITS-4 and M-LCO-4. In Syria, all the mealybugs initially collected as *P*. *comstocki* were identified as *P*. *cryptus*, with haplotypes M-28S-2, M-ITS2-3 and M-LCO-3. In Turkey, the mealybugs were identified as *P*. *comstocki*, with the haplotypes M-28S-1, ITS2-2 and M-LCO-1. In China, the collected mealybugs were identified morphologically as *P*. *comstocki* and displayed haplotypes M-28S-1, M-ITS2-1 and M-LCO-2. The M-LCO-02 haplotype (1.7% divergence from M-LCO-01) was found only in China. Finally, in Japan, all the mealybugs collected were identified as *P*. *comstocki*, with haplotypes M-28S-1, M-ITS2-1, M-LCO-1.

### Survey and characterisation of French and exotic parasitoids infesting *P*. *comstocki*

In total, 314 parasitoid sequences were obtained for P-28S, 179 for P-ITS2, 97 for P-C1 and 59 for P-LCO (Tables 4 and 5). At each site of collection, the most probable host of the characterised parasitoids was determined by identifying a number of mealybugs collected from the site concerned, by DNA sequencing or rapid PCR identification.

**Table 4:** Molecular and morphological characterisation of the mealybug parasitoids collected: morphological identification (the family is provided if not Encyrtidae), haplotypes at 28S, ITS2 and COI (two regions), specimen codes (best preserved slide-mounted specimens in bold).

Identification	Haplotypes				Site codes	Sample codes
	P-28S	ITS2	P-COI	P-LCO		
*Anagyrus* sp. nr *pseudococci (sensu Triapitsyn et al*. *2007)*	P28S-01	PITS2-08-11	PC1-01	PLCO-02	F01, F03, F04, F06, F08, F10, I02, I04, S01	3231, 3264–3266, 3268–3270, **3271–3275**, 3737, **5809**, 5811, **5820**, **5833**, **5848–5850**, **5867**, 5873–5875, **5876**, **5878**, **5881**–5883, **5884**, 5885–**5887**, 5900–5902, 5906–5908, 5915, 5917, 5922–5923, 5933–5935, 5945
	P28S-02	PITS2-14	PC1-03	-	F01, T01	**3215**, 9138
	P28S-03	PITS2-12,13	PC1-02-08	-	F01	3208, **3209–3214**, 3224, **3225**, 3226–3229, **3230**, **3260**
*Leptomastix epona*	P28S-04	PITS2-01-03	PC1-17,18	PLCO-05	F01, F04, F05, F06, F08, F10	**3240–3242**, 3243, 3245, **3246**–3247, 3257, **3262–3263**, **5784**, **5791**, **5793**, 5794–**5795**, 5796, **5797–5798**, **5815–5817**, **5822**, 5823, **5824**, 5827, 5830–5832, 5834, 5836–5838, **5842**, 5851, 5853, **5857**–5858, 5879, 5880, 5898, 5903–5905, 5936, 5952
*Leptomastix histrio*	P28S-05	PITS2-04,05	PC1-19	-	F01, F10	**3244**, **5813**
*Anagyrus fusciventris*	P28S-06	PITS2-15,16	PC1-20	-	F01, F10	3232–3234, **3235**, **3706–3708**, 3709, **3710**, 3711–3712, **3713**, 3735
*Leptomastix dactylopii*	P28S-07	-	-	-	S01	1526, 1528–1536, 5928,** 5929–5932**
*Clausenia purpurea*	P28S-08	-	-	-	S01, T02	1519, 1521–1525, **5910**, 5912–5913, 5918, 5927, 8547–8548, 9428–9429
	P28S-09	PITS2-17,18	PC1-09	PLCO-03	F01, J01, J02	**3652–3653**, **3664–3665**, 3666–3668, 3679–3680, 3691–3693
*Leptomastidea bifasciata*	P28S-10	PITS2-23-27	PC1-12-16	PLCO-04	F01, F04	**3201–3203, 3205, 3207**, 3216–3221, **3222**, 3223, **5845–5847**
	P28S-11	PITS2-25	PC1-13	PLCO-04	F01	**3204**, **3206**
	P28S-12	PITS2-23	PC1-12	-	F01	**3200**
*Acerophagus malinus*	P28S-13	PITS2-06,07	PC1-10,11	PLCO-07-09	F01, F12, J02, J03, T01, T02	**3650**, 3654–3658, **3659**, 3660, **3361–3663**, 3681–3685, **3686–3690**, 9140–9151, 9154–9160, 9162–9164, 9397–9400, 9402–9413, 9422, 9431–9434
Aphelinidae sp.	P28S-14	PITS2-28	PC1-23,24	PLCO-10	F04	**3730, 3732–3733**, 5783, 5786, 5792, 5860–**5861**, 5863–5865
*Pachyneuron* sp. (Pteromalidae)	P28S-15	PITS2-33	-	-	F10	**5800**, **5805**
	P28S-16	-	-	-	S01	**5920**
	P28S-17	PITS2-34	-	-	F10, S01	**5802**, 5924
	P28S-18	-	-	-	F10, S01	**5844**, **5919**, **5921**
*Acerophagus flavidulus*	P28S-19	PITS2-31	-	-	F02, F07, F09, F11	5866, 5938, **5939–5941**, 5943–**5944**, **5953–5954**, 5955
*Thysanus sp*. *(Signiphoridae)*	P28S-20	PITS2-36	-	-	F10	**5804**
*Acerophagus maculipennis*	P28S-21	PITS2-32	-	-	F04, F05	**5855–5856**, 5949–5951
*Cheiloneurus ceroplastis*	P28S-22	PITS2-30	PC1-21,22	PLCO-06	F01, F10	3256, **3258**–**3259**, **3261, 5799**, **5810**, **5818**
	P28S-23	PITS2-29	-	-	F10	**5829**, **5839**–**5840**
*Allotropa burrelli (Platygastridae)*	P28S-24	PITS2-18-22	-	PLCO-01	J01, J02, J03	3644–3645, 3648–**3649**, **3651**, 3669–3673, **3674–3676**, 3677–3678
Pteromalidae	P28S-25				S01	**5909**
Aphelinidae	P28S-26		-	-	F10	**5803**
Pteromalidae	P28S-27	PITS2-35	-		F10	**5843**
	P28S-28	-	-	-	S01	**5911**
*Thomsonisca* sp.	P28S-29	-	-	-	F10	**5828**

**Table 5:** Sequence Genbank accession number for each haplotype found with the markers P-28S, P-ITS2, P-C1 and P-ITS2.

P-28S		P-ITS2		P-C1		P-LCO	
P28S-01	KU499412	PITS2-01	KU499472	PC1-01	KU499448	PLCO-01	KU499514
P28S-02	KU499413	PITS2-02	KU499473	PC1-02	KU499449	PLCO-02	KU499515
P28S-03	KU499414	PITS2-03	KU499474	PC1-03	KU499450	PLCO-03	KU499516
P28S-04	KU499415	PITS2-04	KU499475	PC1-04	KU499451	PLCO-04	KU499517
P28S-05	KU499416	PITS2-05	KU499476	PC1-05	KU499452	PLCO-05	KU499518
P28S-06	KU499417	PITS2-06	KU499477	PC1-06	KU499453	PLCO-06	KU499519
P28S-07	KU499418	PITS2-07	KU499478	PC1-07	KU499454	PLCO-07	KU499520
P28S-08	KU499419	PITS2-08	KU499479	PC1-08	KU499455	PLCO-08	KU499521
P28S-09	KU499420	PITS2-09	KU499480	PC1-09	KU499456	PLCO-09	KU499522
P28S-10	KU499421	PITS2-10	KU499481	PC1-10	KU499457	PLCO-10	KU499523
P28S-11	KU499422	PITS2-11	KU499482	PC1-11	KU499458		
P28S-12	KU499423	PITS2-12	KU499483	PC1-12	KU499459		
P28S-13	KU499424	PITS2-13	KU499484	PC1-13	KU499460		
P28S-14	KU499425	PITS2-14	KU499485	PC1-14	KU499461		
P28S-15	KU499426	PITS2-15	KU499486	PC1-15	KU499462		
P28S-16	KU499427	PITS2-16	KU499487	PC1-16	KU499463		
P28S-17	KU499428	PITS2-17	KU499488	PC1-17	KU499464		
P28S-18	KU499429	PITS2-18	KU499489	PC1-18	KU499465		
P28S-19	KU499430	PITS2-19	KU499490	PC1-19	KU499466		
P28S-20	KU499431	PITS2-20	KU499491	PC1-20	KU499467		
P28S-21	KU499432	PITS2-21	KU499492	PC1-21	KU499468		
P28S-22	KU499433	PITS2-22	KU499493	PC1-22	KU499469		
P28S-23	KU499434	PITS2-23	KU499494	PC1-23	KU499470		
P28S-24	KU499435	PITS2-24	KU499495	PC1-24	KU499471		
P28S-25	KU499436	PITS2-25	KU499496				
P28S-26	KU499437	PITS2-26	KU499497				
P28S-27	KU499438	PITS2-27	KU499498				
P28S-28	KU499439	PITS2-28	KU499499				
P28S-29	KU499440	PITS2-29	KU499500				
		PITS2-30	KU499501				
		PITS2-31	KU499502				
		PITS2-32	KU499503				
		PITS2-33	KU499504				
		PITS2-34	KU499505				
		PITS2-35	KU499506				
		PITS2-36	KU499507				

On the basis of these mealybug identification data, the parasitoids identified were sorted into three categories: (i) parasitoids collected from sites at which only *P*. *comstocki* was detected, (ii) parasitoids collected from sites at which *P*. *comstocki* and other mealybug species were detected, and (iii) parasitoids collected from sites at which only other mealybug species were detected.

In France, at the sites at which only *P*. *comstocki* was observed, six parasitoid taxa of Encyrtidae were identified: *Anagyrus* nr *pseudococci* (*sensu* Triapitsyn *et al*., 2007) (P-28S-01 to P-28S-03, P-C1-02 to P-C1-08, P-ITS2-08/09, P-ITS2-12 to P-ITS2-14, P-LCO-02/11), *Leptomastix epona* (Walker) (P-28S-04, P-C1-17/18, P-ITS2-01 to P-ITS2-03, P-LCO-05), *Anagyrus fusciventris* (Girault) (P-28S-06, P-C1-20, P-ITS2-15/16), *Clausenia purpurea* Ishii (P-28S-09, P-C1-09, P-ITS2-17, P-LCO-03), *Leptomastidea bifasciata* (Mayr) (P-28S-10 to P-28S-12, P-C1-12 to P-C1-16, P-ITS2-23 to P-ITS2-27, P-LCO-04), *Acerophagus malinus* (Gahan) (P-28S-13, P-C1-11, P-ITS2-07, P-LCO-09). A number of other taxa could not be identified with confidence by morphological examination. Two specimens assigned to the species *Leptomastix histrio* (Förster) (P-28S-05, P-C1-19, P-ITS2-04/05) (Hymenoptera: Encyrtidae) were collected, but both were male, making it impossible to be sure about this identification. The hyperparasitoid *Cheiloneurus ceroplastis* Ishii (Hymenoptera: Encyrtidae) was probably present (haplotypes P-28S-22 and P-28S-23, P-C1-21/22, P-ITS-29/30, P-LCO-06). An unidentified member of the Aphelinidae, possibly from the genus *Coccophagus*, was characterised for 28S only (P-28S-26). An unidentified member of the Signiphoridae (P-28S-20, P-ITS2-36) was also collected. A set of probable Pteromalidae hyperparasitoids of the genus *Pachyneuron*, which may contain several species, was also found (P-28S-15/17/18/27; P-ITS2-33 to P-ITS2-35). Finally, one specimen of *Thomsonisca* sp. was collected (P-28S-29).

In France, at sites at which both *P*. *comstocki* and *P*. *viburni* were observed, fixe Encyrtidae taxa were identified: *A*. *nr pseudococci* (P-28S-01, P-ITS2-08), *Leptomastix epona* (P-28S-04, P-ITS2-01, P-ITS2-02), *Acerophagus flavidulus* (Brèthes) (P-28S-19, P-ITS2-31), *Acerophagus maculipennis* (Mercet) (P-28S-21, P-ITS2-32), *Leptomastidea bifasciata* (Mayr) (P-28S10, P-ITS2-26). An unidentified member of the Aphelinidae was also recorded (P-28S-14, P-C1-23/24, P-ITS2-28, P-LCO-10).

In Italy, at sites from which only *P*. *comstocki* was collected, the only identified taxon was *Anagyrus* nr *pseudococci* (P-28S-01, P-C1-01, P-ITS2-08 to P-ITS2-11, P-LCO-02).

In Japan, where only *P*. *comstocki* was detected at the collection sites, three taxa were observed: *Clausenia purpurea* (P-28S-09, P-C1-09, P-ITS2-17/18 and P-LCO-03), *Acerophagus malinus* (P-28S-13, P-C1-10, P-ITS-06 and P-LCO-08) and *Allotropa burrelli* Muesebeck (P-28S-24, P-ITS2-18 to P-ITS2-22, P-LCO-01).

In Turkey, *P*. *comstocki* was the only species found at the collection sites and three species of parasitoids were identified: *Anagyrus* nr *pseudococci* (haplotype P-28S-02), *Clausenia purpurea* (haplotype P-28S-08) and *Acerophagus malinus* (P-28S-13, P-LCO-07) (Hymenoptera: Encyrtidae).

In Syria, where no *P*. *comstocki* was detected, three parasitoid taxa were observed: *Anagyrus* nr *pseudococci* (displaying the haplotype P-28S-01), *Leptomastix dactylopii* (P-28S-07) and *Clausenia purpurea* (P-28S-08) (Hymenoptera: Encyrtidae). A group of specimens could not be identified accurately but probably corresponded to pteromalid hyperparasitoids of the genus *Pachyneuron* (P-28S-16 to P-28S-18, P-28S-25/28).

In China, no parasitoids were detected at the collection site studied.

## Discussion

### Overview of the outcomes of the parasitoid research

The molecular and morphological identification results provided useful information for guiding the decisions made in the biological control programme. In 2009, after a two-year survey in France, no parasitoid species known to be specialist on *P*. *comstocki* had been recorded in apple orchards. This triggered the decision to look for exotic parasitoids attacking *P*. *comstocki*. The mealybug identification obtained from Syria (*P*. *cryptus*) in 2009 led us to discard Syria as a source country for the importation of a natural enemy. The parasitisation of Syrian *P*. *cryptus* by *Clausenia purpurea*, a species also used for the biological control of *P*. *comstocki*, also led us to avoid using this parasitoid in the biological control programme. This finding confirmed published observations of *C*. *purpurea* parasitising several species of mealybugs [33, 34]. In 2010, DNA sequence data from the samples collected in China, revealing the presence of a taxon very closely related to but which maybe different from *P*. *comstocki*, led us to discard the Chinese site as a source of natural enemies for importation. This same year, genetically similar populations of the specialist parasitoid *A*. *malinus* were collected and imported from Japan and unexpectedly collected from ornamental trees in France. We thus decided to start an augmentation programme, making use of the *A*. *malinus* populations already present in France, to avoid the risks associated with the use of exotic material. *C*. *purpurea* was also collected in Japan and France (from ornamental *Morus* spp. only) in 2010. However, the 28S haplotype of these *C*. *purpurea* specimens (P28S-09) was slightly different from that of the *C*. *purpurea* from *P*. *cryptus* in Syria (P28S-08). It therefore remains unclear whether these two taxa are actually the same species. We maintained our decision to exclude this species, which may not be a specialist, from the programme. Again in 2010, populations of *Allotropa burrelli*, known to be a specialist parasitoid of *P*. *comstocki*, were sampled in Japan from *P*. *comstocki* populations apparently similar to the French *P*. *comstocki* populations. *A*. *burrelli* was not detected in the survey performed in France or, more generally, in Western Europe. We therefore decided to introduce this species in the framework of a classical biological control programme. Releases were performed in 2014 and 2015 in Southern France. At the time of the writing of this article, the outcomes of the programme are not known yet.

The complementary sampling carried out in eastern Turkey in 2011 did not identify any new biocontrol agent candidates and, therefore, did not modify our strategy. Instead, the data for the specimens collected revealed the presence of *Clausenia purpurea* of the same haplotype (P28S-08) as the *C*. *purpurea* collected from *P*. *cryptus* in Syria. This provides further support for the hypothesis that *C*. *purpurea* is not a specialist parasitoid of *P*. *comstocki*, instead being able to parasitise two closely related species, *P*. *comstocki* and *P*. *cryptus*, in natural conditions.

### Production of data on *Pseudococcus* parasitoids

In addition to the data used to design the biological control programme for French populations of *P*. *comstocki*, this study generated data concerning the biodiversity of parasitoids of *Pseudococcus* species from various regions of the world. In particular, the DNA data provided insight into the host ranges of several parasitoid species. For example, they revealed that *P*. *comstocki* and *P*. *viburni* were hosts of *Anagyrus* nr *pseudococci* [35], which had previously been collected mainly on *Planococcus* species. The study also provided molecular data for the *Leptomastix epona* populations repeatedly found at sites at which *P*. *comstocki* was likely the only mealybug present (based on mealybug PCR identification results). This material will be used for a thorough revision of the *L*. *epona* group of *Leptomastix*, including the species *L*. *algirica* Trjapitzin and *L*. *flava* Mercet, which may be synonymous, a possibility that should be tested by assessing their interfecundity, as suggested by Anga and Noyes (1999). Our results also suggest that, regardless of its massive introduction into the surveyed region between 2003 and 2006, *A*. *flavidulus* likely does not parasitise *P*. *comstocki*. Indeed, this parasitoid was collected only from sites at which *P*. *viburni* was recorded (regardless of the occurrence of *P*. *comstocki*). Finally, specimens of a range of parasitoid species (from the families Aphelinidae, Encyrtidae, Pteromalidae and Signiphoridae) were occasionally collected. However their low frequency suggests that some of them might have emerged from other hosts located on the plant material brought to the laboratory with the mealybugs. As a consequence, no emphasis was placed on the identification of these specimens.

### Added value of the molecular identification methods

Our consortium benefited from the expertise of taxonomists specialising in the Pseudococcidae (J-F Germain) and their parasitoids (G Delvare and E. Guerrieri), who were able to provide us rapidly with accurate identifications. The use of molecular tools may therefore have been less crucial than in most biological control programmes. Nevertheless, the use of molecular tools was highly advantageous to the programme.

**Fig 1:**
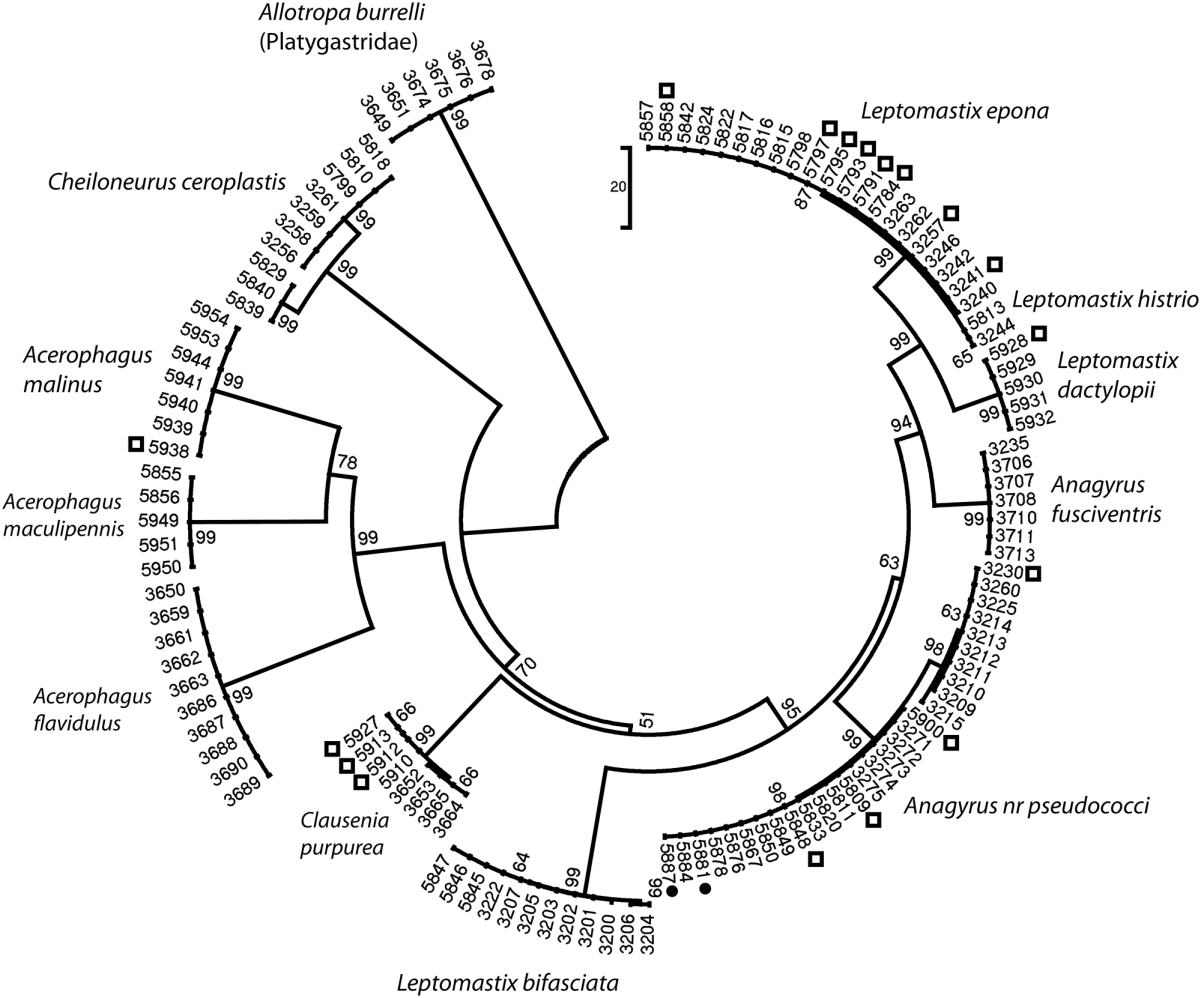
Neighbour-joining tree of 28S haplotypes of Encyrtidae parasitoids (*Allotropa burrelli*, Platygastridae, is used as an outgroup) subjected to morphological examination. Black dots indicate a discrepancy between the molecular and morphological results. Rectangles indicate that the morphological identification, although compatible with the molecular identification, was not conclusive, because the insect was not well preserved or was not of the appropriate sex for identification with the key. The neighbour-joining tree is based on the number of differences. Branch support was calculated by bootstrapping (10,000 iterations).

Firstly, the molecular data facilitated the choice of relevant material for examination and the sharing of complementary information (sequence haplotypes *versus* morphological characters), leading to particularly fruitful collaborations. In total, 145 of the 314 specimens for which DNA sequences were obtained were sent for morphological identification, and comparisons of morphological and molecular data greatly increased the reliability of both types of data (Fig 1).

Secondly, molecular data ensured that identifications were consistent despite the heterogeneity of the material to be identified, in terms of both sample conservation state and development stage. Indeed, it would not have been possible to identify most of the material purely by morphological methods. We estimate that only about 25% of the material collected was suitable for morphological identification. Moreover, even among the specimens selected for morphological identification after DNA sequencing, 24 of the 142 specimens could not be identified and two identifications were inconsistent with the molecular analysis. These difficulties reflect the fact that most of the taxonomic keys for mealybugs and their parasitoids were generally developed for a single sex at a single developmental stage, and an absence of any body parts (legs, antennae) can be problematic.

Thirdly, molecular characterisation ensured that identification was repeatable throughout the programme, providing reliable and consistent identifications on which the team could base their decisions. For example, *C*. *purpurea* and *A*. nr *pseudococci*, which were found at many sites and displayed morphological variability, would have been very difficult to identify in the absence of sequence data revealing similarities and differences between the populations. From a population genetics standpoint, the type of DNA data generated in this study cannot distinguish unambiguously between polymorphic populations within a single species and very closely related taxa (host races, sibling species, etc.). Populations displaying identical haplotypes at several loci cannot therefore necessarily be considered to belong to the same species. However, in practical terms, the probability of such populations being reproductively isolated species with high levels of divergence appears to be low. The data obtained in this study therefore made it easier to take some difficult decisions, such as the decision to rule out *C*. *purpurea* as a specialist candidate biocontrol agent because populations with identical haplotypes were found to parasitize several host species.

Finally, the availability of DNA barcodes for most of the biological material collected made it possible to comply with the requirements of the French government regarding the importation and use of exotic biological material. In particular, the existence of multilocus DNA data for *A*. *burrelli* populations facilitated the obtainment of authorisation for importation from French government services.
